# Optimizing Physiotherapeutic Effects With the Maitland Mobilization Technique to Boost the Functional Capacities of a Rotator Cuff Syndrome Patient: A Case Report

**DOI:** 10.7759/cureus.65341

**Published:** 2024-07-25

**Authors:** Samiksha V Sonone, Deepali S Patil

**Affiliations:** 1 Musculoskeletal Physiotherapy, Ravi Nair Physiotherapy College, Datta Meghe Institute of Higher Education & Research, Wardha, IND

**Keywords:** musculoskeletal physiotherapy, physiotherapy rehabilitation, conservative medical management, maitland mobilization, rotator cuff syndrome

## Abstract

Rotator cuff (RC) muscles give the shoulder joint stability in addition to movement. The case report outlines the physical rehabilitation therapy, condition evaluation, and diagnostic testing that was given to a 62-year-old female field worker who had been complaining of pain in her left shoulder. To improve functional mobility and lessen discomfort, the patient underwent physiotherapy. In this instance, a physical therapy program was put in place to treat rotator cuff syndrome (RCS), enhance range of motion (ROM), and promote long-term recovery. Part of the assessment included a detailed examination of the biomechanics and potential mitigating variables for the persistent problems. The intervention plan's multimodal approach comprised physical therapy, stretching, and strengthening exercises, as well as patient counseling and health management education. Throughout the physiotherapy sessions, the patient's functional mobility improved and their level of discomfort gradually decreased. This case adds to the body of knowledge regarding successful physiotherapy techniques for RC injuries by emphasizing the value of a comprehensive approach to help patients with chronic shoulder pain achieve favorable outcomes. It also highlights how crucial it is to treat chronic RCS with a customized physical therapy program that takes into account the patient's unique preferences and characteristics that can exacerbate the problem.

## Introduction

Rotator cuff (RC) tendinopathy is a predominant reason for pain in the shoulder [1]. The majority of shoulder conditions are thought to be periarticular soft tissue illnesses, which include RC problems. A tear in these RC muscles (commonly supraspinatus) results in shoulder impingement, decreased range of motion (ROM), and joint pain [2]. Stabilizing the glenoid humeral head and facilitating some glenohumeral joint motion are the two main functions of RC muscles. There are two sorts of causes: internal and extrinsic, and traumatic and non-traumatic [3]. One may experience acute, chronic, or acute-on-chronic RC injuries [4]. The drop arm test is used to detect the tear in the supraspinatus muscle; the subscapularis is evaluated using the bear hug test, belly press, and belly-off sign [5]. The majority opinion is that when the humeral head is dynamically stabilized onto the glenoid fossa, the RC muscles and tendons are engaged synchronously and uniformly throughout shoulder movement [6].

Individuals with degenerative RC injuries frequently complain of sleep disturbance, which is clinically evident in more than 85% of these cases [7]. Conservative therapy may be useful for patients with small tears (less than 10 mm). [8]. Individuals with big or major tears with persistent irreversible RC tears who are older than 65 years old should have nonsurgical treatment. There is a 25% chance of progressive tears with these tears, and there is little chance of long-term, permanent damage [9]. A shoulder brace might be recommended if the pain is making it difficult to go about your everyday activities and is made worse by movement [10]. Among nonsurgical treatments for RC problems, musculoskeletal physiotherapy, which comprises manual therapies, seems to have the strongest backing. It also re-establishes proper glenohumeral joint kinematics. Exercise training will also establish the stability, mobility, and proprioception of the shoulder [11]. It is commonly stated that both physical therapy and tendon repair are effective treatments for small to medium-sized RC problems [12].

Joint mobilization (JM) is one manual treatment method that is mostly used to improve the ROM and reduce discomfort. Low-velocity, repetitive passive motions with varying amplitudes make up oscillatory JM methods. The patient regulates the rate and amplitude of these, which are applied at different sites within the patient's permitted joint ROM [13]. The procedures are graded based on amplitude and are intended to recreate the motion of glide, roll, and spin between joint surfaces. While grades ll & lV of the Maitland mobilization techniques are mostly employed as stretching exercises, grades l and ll are generally utilized to treat joints limited by discomfort [14].

## Case presentation

Patient information

**Figure 1:**
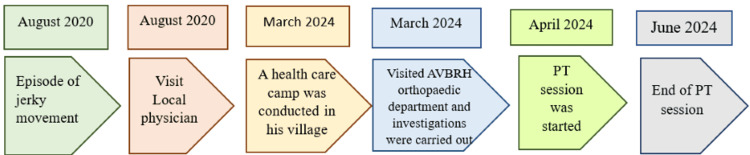
Timeline of events AVBRH: Acharya Vinoba Bhave Rural Hospital; PT: physiotherapy

**Figure 2:**
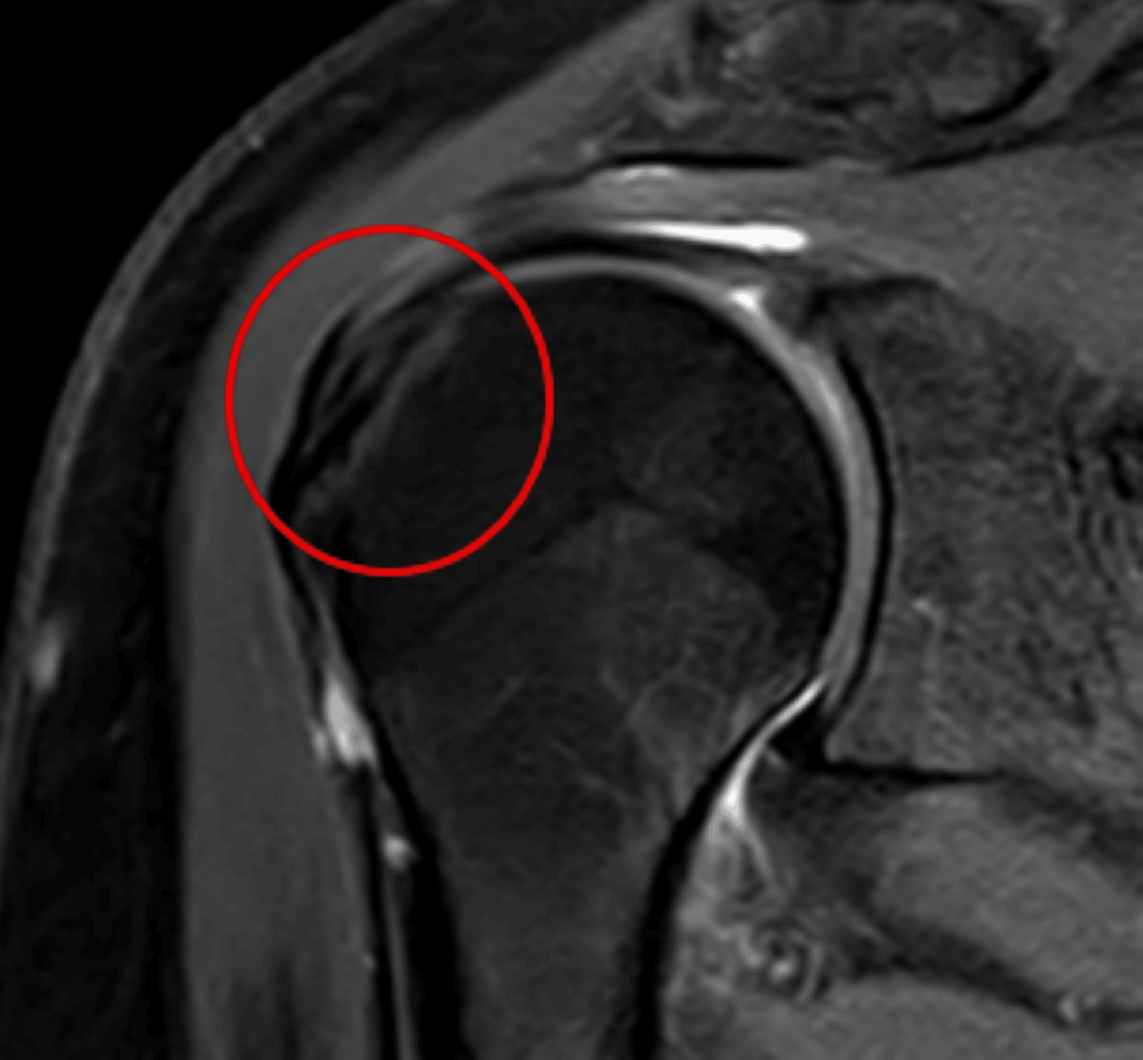
The MRI of the left shoulder joint signifies a partial thickness tear in the rotator cuff muscle (circle)

A 62-year-old female who works as a field worker by occupation and has left-hand dominance came to the physiotherapy department complaining of discomfort in her left shoulder that had been troubling her for the previous four years. It was found after reviewing the patient's long medical history that she had been dealing with pain and restricted ROM in her left shoulder for four years. The patient went on a religious pilgrimage in 2020 and climbed the hill with the help of a stick. She put additional pressure on the stick while climbing, which caused her to have left shoulder pain. Gradually, her pain started to get aggravated, which led her to visit a local physician nearby. There, she was prescribed medication, but her pain was not relieved completely. Years passed, and she neglected her symptoms. In 2024, a health check-up camp was conducted in her village. She was referred to the orthopedic department, where she was suggested for investigations including X-rays and an MRI, which revealed a partial RC tear in her left shoulder. In Figure 1, a timeline of events is mentioned, and in Figure 2, MRI images are presented. Zerodol SP and Pan 40 mg were given to the patient to benefit from inflammation and pain, and the patient was then referred to the physiotherapy unit for further management. 

Clinical findings

The patient sounded alert and focused. The patient gave their consent after being properly informed. There was a physical assessment. Both a supine and a sitting position were used to examine the patient. Palpation revealed no discernible edema and rather mild swelling. The left shoulder was noted to be grade 2 tender. The discomfort was dull, and hurting which started slowly and got worse over time. It was made worse by shoulder flexion and abduction, as well as by overhead activity and rotating motions like turning on and off the tap. On the visual analog scale (VAS), the pain assessment for pre-rehabilitation was 8.9/10 on activity and 4.5 on rest, whereas post-rehabilitation was VAS 4.3 on activity and 1.3 on rest. On clinical evaluation, belly press, lift-off test, and empty can test, all tests were positive. The ROM of the left shoulder was reduced as compared to the right side; it is mentioned in Table 1 (before the physiotherapy intervention) and Table 2 (post-physiotherapy intervention). The manual muscle testing for the left shoulder was also found to be reduced as compared to the right side mentioned in Table 3 (before the physiotherapy intervention) and Table 4 (post-physiotherapy intervention).

**Table 1:** Range of motion before the physiotherapy intervention

Movements of the shoulder joint	Left		Right	
	Active	Passive	Active	Passive
Flexion	0-100 degrees	0-117degrees	0-165 degrees	0-170 degrees
Extension	0-35 degrees	0-40 degrees	0-57 degrees	0-60 degrees
Abduction	0-105 degrees	0-112degrees	0-175 degrees	0-178 degrees
Adduction	0-27 degrees	0-35 degrees	0-45 degrees	0-50 degrees
Internal rotation	0-45 degrees	0-49 degrees	0-77 degrees	0-80 degrees
External rotation	0-40 degrees	0-45 degrees	0-80 degrees	0-85 degrees

**Table 2:** Range of motion post physiotherapy intervention

Movements of the shoulder joint	Left		Right	
	Active	Passive	Active	Passive
Flexion	0-160 degrees	0-168 degrees	0-175 degrees	0-180 degrees
Extension	0-45 degrees	0-53 degrees	0-57 degrees	0-60 degrees
Abduction	0-150 degrees	0-160 degrees	0-175 degrees	0-178 degrees
Adduction	0-30 degrees	0-40 degrees	0-45 degrees	0-50 degrees
Internal rotation	0-55 degrees	0-63 degrees	0-77 degrees	0-80 degrees
External rotation	0-60 degrees	0-70 degrees	0-80 degrees	0-85 degrees

**Table 3:** Manual muscle testing (strength) performed before the physiotherapy intervention

Joint	Movement	Left	Right
Shoulder	Flexion	02/05	05/05
	Extension	02/05	05/05
	Abduction	02/05	05/05
	Adduction	02/05	05/05
	Internal rotation	02/05	05/05
	External rotation	02/05	05/05

**Table 4:** Manual muscle testing after the physiotherapy intervention

Joint	Movement	Left	Right
Shoulder	Flexion	03/05	05/05
	Extension	03/05	05/05
	Abduction	03/05	05/05
	Adduction	03/05	05/05
	Internal rotation	03/05	05/05
	External rotation	03/05	05/05

Therapeutic intervention

**Figure 3:**
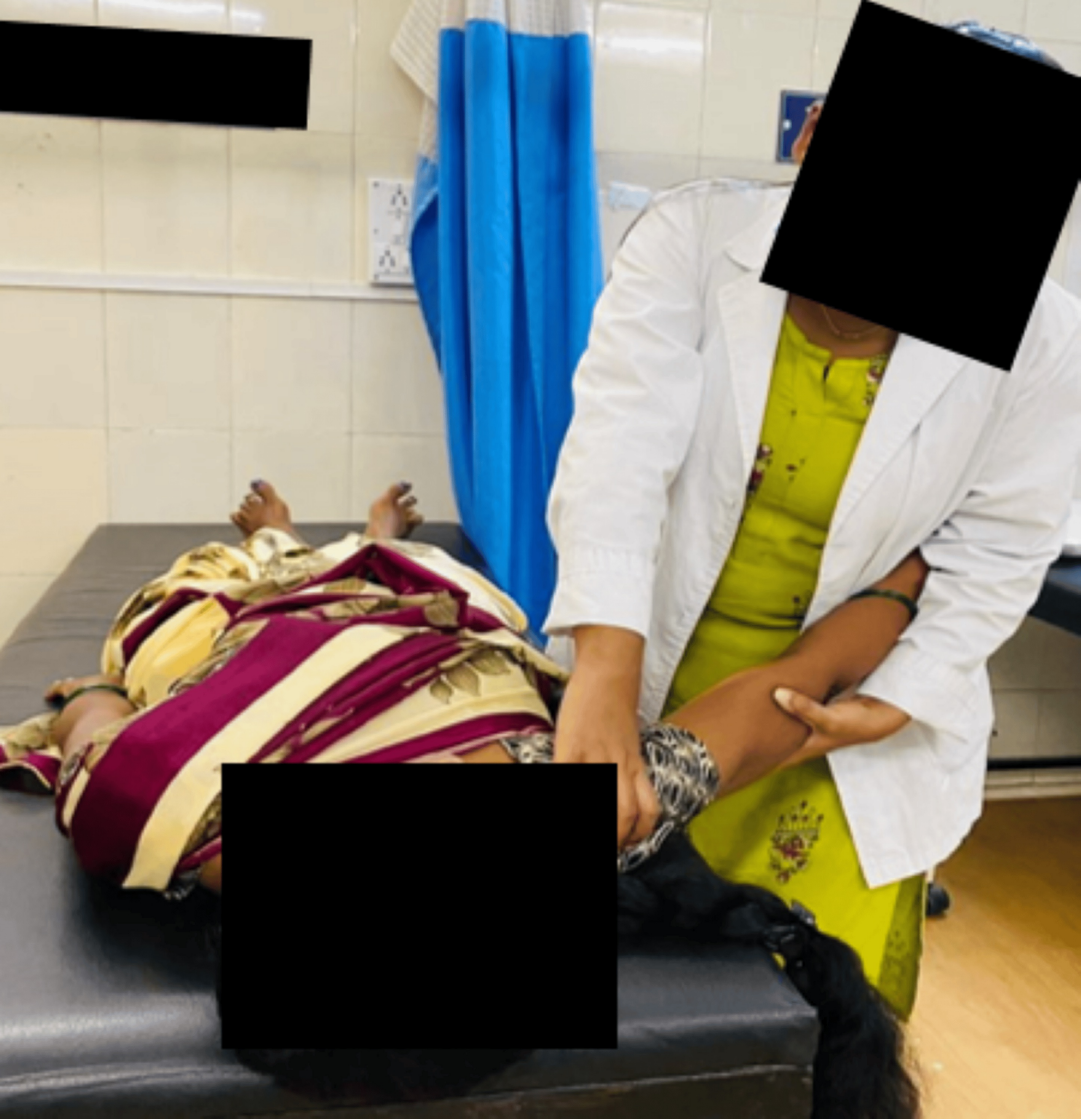
Maitland mobilization for the glenohumeral joint: caudal glide in the resting position

A rehabilitation training program was created to alleviate discomfort, enhance mobility, and facilitate long-term recovery. The regimen was designed for six weeks to improve the quality of life, minimize shoulder joint impairment, restore motion, relieve pain, and promote general shoulder care. In Table 5, therapeutic intervention is displayed, and in Figure 3, the Maitland mobilization technique for the glenohumeral joint for caudal glide is mentioned.

**Table 5:** A summary of the therapeutic interventions

Sr. No	Physiotherapy goals	Therapeutic intervention	Treatment regime/dosages
1.	Counseling of the patient	Educate the patient concerning signs, symptoms, causes, and nature of the injury	Instruct patients on how to avoid injuries and how to properly warm up and cool down
2.	To decrease pain	Ultrasound (US) and transcutaneous electrical nerve stimulation (TENS)	We used US at 1.5 MHz for seven minutes and TENS at 100 Hz for 10 minutes.
3.	For improving muscular strength	Exercises to strengthen the right rotator cuff (RC) isometric: wall push-ups in the scapular plane	From the first day of physiotherapy rehabilitation, twice daily, it was recommended to accomplish two sets of 10 repetitions of wall pushups and active mobility exercises of the left shoulder joint with three fingers and maximal resistance over the wrist.
4.	Progressive strength training exercises	Theraband exercises were done with a yellow color band; later it progressed to a red color.	Ten repetitions of two sets, starting with a five-second hold and working up to 10-second holds
5.	To improve range of motion (ROM)	Manual therapy, which includes mobilization, glenohumeral mobilization posterior, and caudal glide	One manual therapy technique called joint mobilization (JM) is primarily used to increase the ROM and lessen discomfort. Optional JM techniques consist of low-speed, repeated passive movements with different amplitudes. The speed and amplitude of these are controlled by the patient and are applied at various points within the patient's allowed range of motion. Each glide for 30 seconds to one minute.

Outcome measures

Outcomes prior to physiotherapy were assessed on the first day of treatment, and post-physiotherapy outcomes were assessed following a six-week physiotherapy intervention. Using the VAS, the patient's observed pain was assessed. The ROM was measured with a goniometer. The assessment of physical strength was done using the manual muscle test. Disability of the arm, shoulder, and hand were utilized to rate and assess the shoulder's level of disability. Additionally, the Short Form-36 (SF-36) was utilized to assess quality of life. The results demonstrate a statistically significant improvement in the outcome measures following the physiotherapy regimen. Table 6 lists the resultant measures.

**Table 6:** Outcome measures before and after physiotherapy intervention SF-36: Short Form (36); PF: Physical Function; RP: role physical; BP: Bodily Pain; GH: General Health; VT: Vitality; SF: Social Function: RE: Role Emotional; MH: Mental Health; DASH: The Disabilities of the Arm, Shoulder, and Hand Index; ROM: Range of Motion; VAS: Visual Analog Scale VAS scale interpretation: Scored on a 1 to 10 range, 0 indicating no pain and 10 indicating the worst pain; SF-36 scale interpretation: Scored on a 0 to 100 range, a higher score represents better health; DASH scale interpretation: Scored on a 0 to 100 range, a higher score indicates greater disability.

Outcome measures	Before physiotherapy intervention	Post- physiotherapy intervention
SF-36- MH	76%	80%
SF-36-RE	53%	68%
SF-36-SF	60%	87%
SF-36-VT	62%	72%
SF-36-GH	62%	68%
SF-36-BP	30%	50%
SF-36-RP	26%	70%
SF-36-PF	67%	83%
Shoulder flexion	120 degrees	160 degrees
Shoulder extension	35 degrees	45 degrees
Shoulder abduction	120 degrees	150 degrees
Shoulder adduction	27 degrees	30 degrees
Shoulder internal rotation	45 degrees	55 degrees
Shoulder external rotation	40 degrees	60 degrees
DASH	50	17
VAS (on rest)	4.5	1.3
VAS (on activity)	8.9	4.3

## Discussion

A variety of RC pathologies are collectively referred to as RC disease, including bursitis in the subacromial/subdeltoid (SASD) region, calcification, shoulder impingement syndrome, and rotator cuff tendinopathy [15]. Conservative management strategies for RC injuries include non-pharmacological options such as acupuncture, activity modification, physical therapy, and electrotherapies, as well as the use of pharmaceuticals such as drugs [16].

In the nonoperative care of RC injury, physical therapy proved to be beneficial, according to a study by Edwards et al. Understanding that a person's responsiveness and symptom reappearance would regulate the probability of a fruitful exercise rehabilitation program was critical. Exercises using a pendulum to increase shoulder joint ROM stretches with door frames and towels to strengthen muscles, and exercises involving scapular protraction and retraction to strengthen the scapular stabilizers were all part of the rehabilitation procedure used in this study [17].

These exercises were tried on our patient as well, and it was discovered that they helped with ROM, muscle strength, and flexibility. According to Dickinson et al., there is a suggestion in the literature that suggests cryotherapy, also known as ice therapy, may be helpful for pain relief, better sleep, lowering drug dosages, and increasing engagement in therapeutic exercises. Even though continuous cryotherapy facilities are more modern, an Ace bandaged ice pack can be just as effective at a lower cost [18]. Cryotherapy was also used and proven to be successful. Conti et al.'s research indicates that postoperative physiotherapy is crucial for controlling secondary problems and facilitating patients' activities of daily living, both of which improve quality of life [19].

Zhang's studies indicate that the most effective approach to managing shoulder joint dysfunction following RC injury repair is to combine scapula training exercises with routine rehabilitation therapy. However, there is evidence that increasing the number of scapula training exercises improves pain outcomes, the range of activities, and total functional assessment scores [20].

Research by Ellenbecker et al. states that to restore a suitable ROM, RC strength, and scapular stability, clinical rehabilitation combines essential physical procedures with evidence-based rehabilitation approaches [21].

## Conclusions

For patients with rotator cuff syndrome (RCS), a desirable rehabilitation regimen of six weeks was proposed, which involves a positive long-term recovery. In this case study, RC tendinopathy is described along with the patient's physical therapy recovery. Her physical therapy sessions matched her needs and were satisfying. The comprehensive physiotherapy program, which relieved the pain associated with RCS and included targeted exercises, stretching exercises, and manual treatments, generally improved her functional mobility.

The patient indicated improvements in her upper limb functional activity in range of motion, strength of rotator cuff muscles, functional independence, and her activities of daily living. This case demonstrates the vital role that physiotherapists play in enhancing the quality of life for individuals with musculoskeletal issues and emphasizes the need for customized rehabilitation plans adapted to each patient's specific needs. In order to treat and manage problems like RCS holistically and to help patients recover and return to maximum health, rehabilitation is essential. We believe that this case report contributes to our understanding of the most effective approaches to treating these patients.
